# Thiotaurine Attenuates TNF-α-Induced Inflammation in Human Chondrocytes via NF-κB Pathway Suppression and Thiol-Dependent Persulfidation

**DOI:** 10.3390/ijms262010208

**Published:** 2025-10-20

**Authors:** Alessia Mariano, Irene Bigioni, Alessia Baseggio Conrado, Antonio Francioso, Anna Scotto d’Abusco, Mario Fontana

**Affiliations:** 1 Department of Biochemical Sciences, Sapienza University of Rome, P.le Aldo Moro, 5, 00185 Roma, Italy; alessia.mariano@uniroma1.it (A.M.); irene.bigioni@uniroma1.it (I.B.); anna.scottodabusco@uniroma1.it (A.S.d.); 2 Department of Experimental Medicine and Rheumatology, William Harvey Research Institute, Queen Mary University of London, Charterhouse Square, London EC1M 6BQ, UK; a.baseggioconrado@qmul.ac.uk; 3 Department of Bioscience and Technology for Food, Agriculture and Environment, University of Teramo, Via R. Balzarini 1, 64100 Teramo, Italy; afrancioso@unite.it

**Keywords:** sulfane sulfur, Thiotaurine, H_2_S metabolism, osteoarthritis, joint inflammation, human primary chondrocytes

## Abstract

Thiotaurine (2-aminoethane thiosulfonate) is a naturally occurring sulfur-based compound featuring a thiosulfonate group, enabling it to act as a biologically relevant donor of hydrogen sulfide (H_2_S) through thiol-dependent persulfidation. H_2_S levels are known to be reduced in individuals with osteoarthritis, where it plays roles in modulating inflammation, oxidative stress, and pain. This study investigated the anti-inflammatory effects of Thiotaurine in human primary chondrocytes exposed to a pro-inflammatory cytokine. Cells were pre-treated with Thiotaurine prior to stimulation with TNF-α, and the expression levels of key interleukins were assessed at both the mRNA and protein levels. TNF-α stimulation led to upregulation of IL-6, IL-8, and IL-1β, which was significantly attenuated by Thiotaurine pre-treatment. Additionally, immunofluorescence analysis showed that Thiotaurine inhibited the phosphorylation and nuclear translocation of p65, indicating suppression of NF-κB pathway activation. Persulfide detection assays confirmed an increase in intracellular persulfide levels following Thiotaurine treatment. In summary, due to its anti-inflammatory activity and ability to release H_2_S, Thiotaurine emerges as a promising and potentially safe therapeutic option for osteoarthritis and other inflammation-related conditions.

## 1. Introduction

Hydrogen sulfide (H_2_S) is now recognized as a critical gasotransmitter, alongside nitric oxide (NO) and carbon monoxide (CO), involved in diverse physiological processes, including vasodilation, neuromodulation, and inflammation regulation [1,2,3,4,5,6]. Traditionally known for its toxicity, H_2_S has gained attention over recent decades for its role in cellular signalling and homeostasis [7]. Endogenously produced through enzymatic pathways involving cystathionine β-synthase (CBS), cystathionine γ-lyase (CSE), and 3-mercaptopyruvate sulfurtransferase (MST), H_2_S exerts potent cytoprotective, antioxidant, and anti-inflammatory effects [8,9,10].

Among inflammatory and degenerative conditions, osteoarthritis (OA) stands out for its high prevalence and significant social burden. OA is characterized by progressive degradation of articular cartilage, synovial inflammation, and chronic pain, often driven by oxidative stress and pro-inflammatory cytokines such as IL-1β, TNF-α, and IL-6 [11,12,13]. Modulating these molecular pathways through redox-sensitive signaling has emerged as a potential therapeutic strategy. The Nuclear Factor kappa-light-chain-enhancer of activated B cells (NF-κB) is a transcription factor family, mainly involved in cytokine production in OA. The NF-κB family has several factors, among them p65, which is present in the cytoplasm of cells inhibited by factors such as IκBα. After cell stimulation, p65 can migrate into the nuclei, and, after phosphorylation, it activates the transcription of pro-inflammatory mediators [14,15].

H_2_S has a key role in joint health, regulating bone homeostasis and intracellular signaling in various metabolic pathways in all cell joints. It has been demonstrated that H_2_S administration in OA patients can alleviate inflammation, oxidative stress, and pain, thereby counteracting OA progression [6,16].

In this context, Thiotaurine (2-aminoethane thiosulfonate), a naturally occurring sulfur-containing compound and member of the taurine family, has attracted attention for its ability to act as a biologically relevant sulfur donor [17,18]. Structurally similar to taurine and hypotaurine, Thiotaurine uniquely contains a sulfane sulfur moiety, which can be transferred to biological acceptors such as glutathione, leading to H_2_S production [19]. This release occurs under physiological conditions, making Thiotaurine an appealing candidate for targeted H_2_S delivery.

From a historical perspective, the presence of Thiotaurine in mammalian systems was first demonstrated by Cavallini, De Marco, and Mondovì in 1959, who provided chromatographic evidence of its occurrence in the urine of rats fed a cystine-enriched diet [17]. This finding confirmed the *in vivo* production of Thiotaurine as a metabolite of the sulfur amino acid pathway. The identification was supported by chemical synthesis and cyanolysis experiments, which confirmed that the compound was indeed the thiosulfonate analogue of taurine. This discovery marked the first demonstration of Thiotaurine as a naturally occurring component in biological fluids, laying the foundation for subsequent research on its metabolic and signaling roles [20,21,22].

Biochemically, Thiotaurine is synthesized endogenously via multiple routes, including sulfurtransferase-catalyzed reactions involving hypotaurine and mercaptopyruvate. The thiosulfonate group of Thiotaurine, bearing the reactive sulfane sulfur, not only allows H_2_S release but also contributes to its inclusion in the broader group of reactive sulfur species (RSS), increasingly implicated in cellular redox signaling. This makes Thiotaurine not only a storage form of H_2_S but also a key intermediate in the persulfidation of proteins, a post-translational modification critical for regulating enzyme function and inflammatory responses.

Experimental studies have demonstrated that Thiotaurine exerts antioxidant and anti-inflammatory effects by modulating neutrophil activation, preventing apoptosis, and reducing oxidative stress. It has been shown to inhibit caspase-3 activation and influence protein expression involved in energy metabolism and redox balance, such as GAPDH, particularly in activated human neutrophils [21,22].

The present study aims to further explore the biological activities of Thiotaurine in inflamed chondrocytes and elucidate its mechanisms of action in modulating inflammation and H_2_S-mediated signaling.

## 2. Results

### 2.1. Cellular Viability After Thiotaurine Treatment

**Figure 1:**
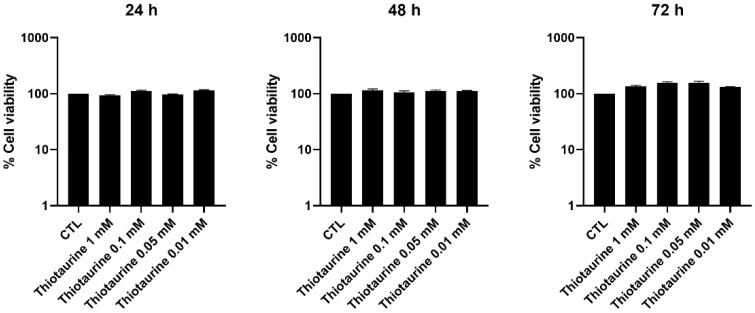
Cell viability was assessed by the MTS colorimetric method, and HPCs were treated with four concentrations, 1 mM, 0.1 mM, 0.05 mM, and 0.01 mM of Thiotaurine, for 24, 48, and 72 h. Cell viability of treated samples was normalized to the untreated cells (CTL).

The effect of Thiotaurine treatment on HPC cell viability was assessed by MTS, finding that no analyzed concentration was detrimental at 24 h, 48 h, and 72 h (Figure 1). Two concentrations, 0.1 mM and 0.05 mM, were used for further experiments.

### 2.2. Effect of Thiotaurine on Inflammatory Mediators

#### 2.2.1. Interleukin Modulation

**Figure 2:**
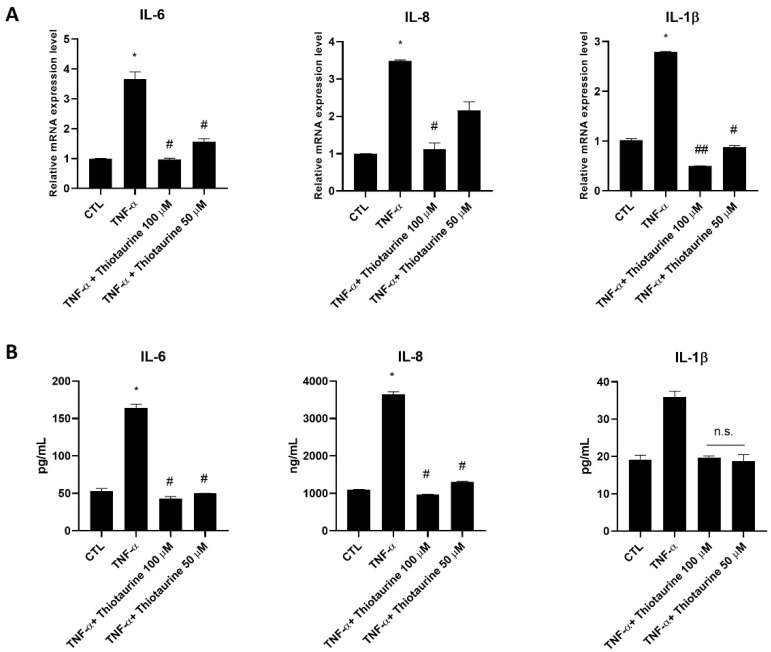
Effects of Thiotaurine on interleukin expression levels and secretion under TNF-α stimulus. (**A**) Cells were left untreated (CTL), or stimulated with 10 ng/mL TNF-α for 30 min, or treated with 100 μM and 50 μM concentrations of Thiotaurine for 1 h and then stimulated with 10 ng/mL TNF-α for 30 min. After treatments, mRNA was extracted and analyzed by RT-PCR. IL-6, IL-8, and IL-1β mRNA levels were reported as relative mRNA expression levels with respect to 18S mRNA (2^−ΔΔCt^ method). (**B**) Cells were left untreated (CTL) or stimulated with 10 ng/mL TNF-α for 1 h or treated with 100 μM and 50 μM concentrations of Thiotaurine for 1 h and then stimulated with 10 ng/mL TNF-α for 1 h. After treatments, the supernatant media were collected and analyzed by ELISA. The results are reported as ng/mL or pg/mL, and all the results are expressed as mean ± standard deviation (SD) of data obtained by three independent experiments. * *p* < 0.05 TNF-α vs. CTL; # *p* < 0.05 Thiotaurine vs. TNF-α; and ## *p* < 0.01 Thiotaurine vs. TNF-α; not significant (n.s) Thiotaurine vs. TNF-α.

To verify the anti-inflammatory activity of Thiotaurine, HPCs were stimulated with TNF-α to produce inflammatory conditions after pre-treatment with two Thiotaurine concentrations. The stimulation with TNF-α for 30 min showed a statistically significant increase in pro-inflammatory interleukins IL-6, IL-8, and IL-1β at the mRNA level compared to untreated cells (CTL). The pre-treatment with Thiotaurine for 1 h was able to counteract the pro-inflammatory interleukin production, restoring the mRNA basal level (Figure 2A). Only 50 μM Thiotaurine was not able to restore the basal level of IL-8 at the mRNA level, even if a decrease was appreciated compared to TNF-α stimulation (Figure 2A). To confirm the results obtained at the mRNA level, an Enzyme-linked ImmunoSorbent Assay (ELISA) was performed on cell media of the untreated and treated cells. The results on IL-6 and IL-8 secretion were perfectly in line with the findings obtained at the mRNA level. In this case, the 50 μM Thiotaurine concentration was effective to statistically decrease IL-8 production (Figure 2B). Regarding IL-1β secretion, the TNF-α stimulation was not statistically significant compared to the CTL, while the two Thiotaurine concentrations decreased the IL-1β production at the CTL level (Figure 2B).

#### 2.2.2. Effect on p65 Phosphorylation (p-p65) and Expression

**Figure 3:**
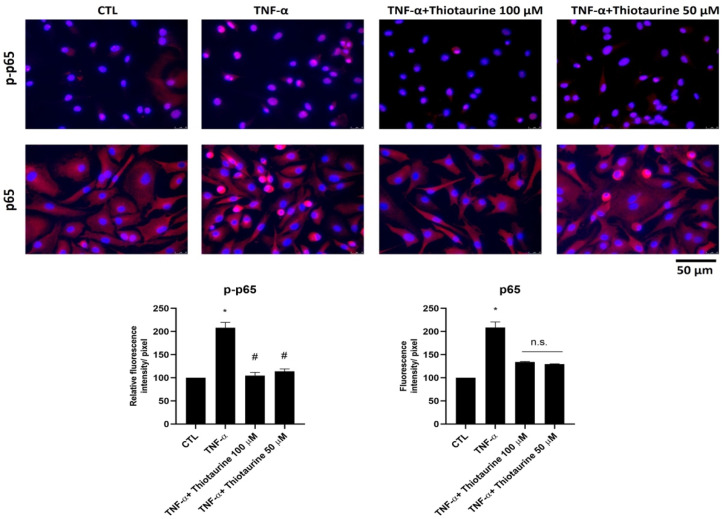
Effects of Thiotaurine on phosphorylation (p-p65) and expression of p65. Cells were treated with 100 μM and 50 μM Thiotaurine for 1 h and then stimulated with 10 ng/mL TNF-α for 10 min and then analyzed by immunofluorescence using anti-p-p65 and p65 primary antibodies and Alexa Fluor 594 (red) secondary antibody. Nuclei were stained with DAPI (original magnification 40×). The bar graph represents the pixel intensities in the region of interest, obtained by ImageJ. The p-p65 intensity has been normalized with respect to total p65. The results are expressed as mean ± SD of data obtained by three independent experiments. * *p* < 0.05 TNF-α vs. CTL; # *p* < 0.05 Thiotaurine vs. TNF-α. Not significant (n.s.) Thiotaurine vs. TNF-α.

**Figure 4:**
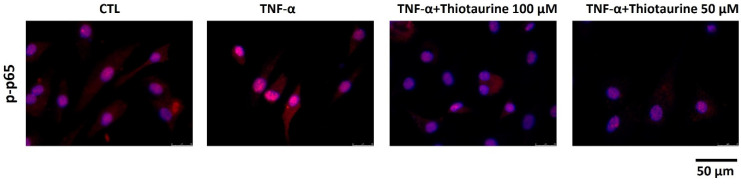
Effects of Thiotaurine on p65 phosphorylation (p-p65). Cells were treated with 100 μM and 50 μM Thiotaurine for 1 h and then stimulated with 10 ng/mL TNF-α for 10 min and then analyzed by immunofluorescence using anti-p-p65 primary antibody and Alexa Fluor 594 (red) secondary antibody. Nuclei were stained with DAPI (original magnification 63×).

The pro-inflammatory intracellular pathways involve several mediators, among them NF-κB. NF-κB has several isoforms, such as p65, which is particularly stimulated in OA disease. In cells, p65 is kept inactive in the cytoplasm by IκBα. Upon pro-inflammatory stimulation, p65 becomes phosphorylated (p-p65) and translocates to the nucleus to activate gene transcription. To verify whether Thiotaurine had an effect on the NF-κB pathway, we analyzed the phosphorylation and nucleus migration of p65 under TNF-α stimulation. We found that, following stimulation, p65 was more phosphorylated (p-p65) and localized mainly into the nuclei (Figure 3). The pre-treatment with 50 μM and mainly with 100 μM Thiotaurine decreased both p65 phosphorylation (Figure 3 upper line) and p65 production (Figure 3 bottom line). The inhibition of phosphorylation was statistically significant, whereas the decrease in total p65 was not, as reported in the densitometric analysis in Figure 3. This inhibition of p65 phosphorylation (p-p65) was also reported in Figure 4 at a higher magnification (63×).

### 2.3. In Vitro Effect of Thiotaurine on Persulfide Formation

To verify the ability of Thiotaurine to stimulate persulfide formation, we performed a fluorescent experiment in a living HPC cell culture.

The HPCs were left untreated (CTL) or treated for 24 h with 100 µM or 50 µM of Thiotaurine. The cells were then incubated with EZ-Link™ Iodoacetyl-PEG2-Biotin, a reagent that is able to bind both sulfide and persulfide molecules present inside cells, and visualized using Alexa Fluor 488-Streptavidin.

**Figure 5:**
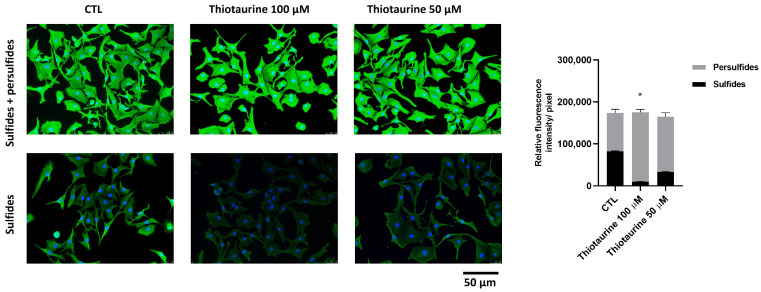
Effects of Thiotaurine on sulfide and persulfide production. Cells were treated with 100 μM and 50 μM Thiotaurine for 24 h and then incubated with EZ-Link™ Iodoacetyl-PEG2-Biotin, reagent able to bind both sulfide and persulfide molecules present inside cells and visualized using Alexa Fluor 488-Streptavidin (green). To visualize only sulfide species, cells were treated with DTT. Nuclei were stained with DAPI (original magnification 40×). The bar graph represents the pixel intensities in the region of interest, obtained by ImageJ. The black bars represent sulfide species; gray bars represent persulfide species obtained by difference between total and sulfide species. The results are expressed as mean ± SD of data obtained by three independent experiments. * *p* < 0.05 persulfide in 100 μM Thiotaurine-treated cells vs. CTL.

To visualize sulfide only, cells were incubated with DTT. The differences between the integrated fluorescence density values obtained in sulfide and persulfide wells and sulfide wells indicated the persulfide expression. The expression of sulfide and persulfide species was not influenced by the Thiotaurine treatment at either of the used concentrations. After the DTT treatment, the persulfide species were lost due to sulfur bond cleavage, and only sulfide species remained fluorescent. The Thiotaurine treatment caused a decrease in sulfide species, as can be seen in Figure 5 (bottom line); this means that the persulfide species were higher in Thiotaurine-treated cells compared to the CTL, and 100 μM was more effective than the 50 μM concentration (Figure 5).

## 3. Discussion

This study investigated the anti-inflammatory and H_2_S-donating properties of Thiotaurine in human primary chondrocytes (HPCs) under pro-inflammatory conditions. Our findings support the hypothesis that Thiotaurine exerts a protective role against inflammatory activation in OA-related cellular models through mechanisms involving cytokine modulation, NF-κB pathway inhibition, and enhancement of persulfidation.

Firstly, Thiotaurine demonstrated no cytotoxic effects on HPCs at the tested concentrations, establishing its suitability for further biological testing. Upon TNF-α stimulation, a well-established *in vitro* model of inflammatory response, we observed significant upregulation of pro-inflammatory mediators IL-6, IL-8, and IL-1β. Pre-treatment with Thiotaurine effectively reversed this response at both the mRNA and protein levels, particularly at a 100 µM concentration, which was the most consistent in reducing cytokine expression. Importantly, this anti-inflammatory effect of Thiotaurine exhibited a clear dose-dependent trend, with higher concentrations producing more pronounced reductions in pro-inflammatory mediator levels, further supporting its potential therapeutic relevance. These results align with previous observations in immune cells such as neutrophils, where Thiotaurine demonstrated the capacity to downregulate inflammatory mediators and oxidative stress markers.

A key mechanistic insight emerged from the analysis of the NF-κB signaling cascade. TNF-α-induced phosphorylation of the p65 subunit and its nuclear translocation were significantly inhibited by Thiotaurine, suggesting that its anti-inflammatory activity partly stems from the suppression of NF-κB activation. This is of particular relevance given the central role of p65 in OA progression, where sustained NF-κB signaling promotes matrix degradation through the over-production of degrading enzymes, such as metalloproteases, and chondrocyte apoptosis [23]. Overall, these mechanisms promoted extracellular matrix degradation, leading to joint destruction [24,25]. Moreover, Thiotaurine showing the inhibition of p65 phosphorylation further supports a broader modulatory effect on inflammatory transcriptional responses.

The increase in intracellular persulfide species, accompanied by a decrease in free sulfide levels, indicates enhanced persulfidation activity, confirming that Thiotaurine works as a biologically active sulfur donor [26,27]. This process has been recognized as a critical post-translational modification capable of regulating protein function, redox signaling, and cellular resilience against oxidative stress. Since the balance between sulfide and persulfide pools is essential in redox homeostasis, the ability of Thiotaurine to tilt this balance toward persulfidation is a relevant therapeutic feature, distinguishing it from less selective H_2_S donors [28,29,30]. Beyond its role in redox regulation, persulfidation has emerged as a key modulator of inflammatory pathways. It can inhibit the NF-κB signaling cascade by modifying cysteine residues on key regulatory proteins, thereby reducing the transcription of pro-inflammatory cytokines such as TNF-α, IL-1β, and IL-6 [31,32,33,34]. Additionally, persulfidation can activate the Nrf2 pathway, enhancing the expression of cytoprotective and anti-inflammatory genes, which further supports cellular defense against inflammation [35]. Therefore, the ability of Thiotaurine to increase intracellular persulfide levels may contribute to its anti-inflammatory properties through both the inhibition of pro-inflammatory signaling and reinforcement of antioxidant defenses. Recent studies have found that reactive intermediates other than H_2_S react with thiols to generate persulfides [36,37]. Some of these reactive intermediates have been identified among the sulfane sulfur compounds [38,39]. In this context, Thiotaurine, with its sulfane sulfur moiety, represents a biologically relevant H_2_S donor in the persulfidation reactions of protein cysteine (reaction 1).

RSO_2_SH + RSH → RSO_2_H + RSSH (1)

Taken together, our data demonstrates that Thiotaurine exerts multiple layers of control over inflammatory responses in chondrocytes, acting both at the transcriptional level through NF-κB inhibition and at the post-translational level via persulfide formation. These actions may confer efficacy in managing chronic joint inflammation compared to conventional anti-inflammatory agents.

## 4. Materials and Methods

### 4.1. Thiotaurine Synthesis

Thiotaurine was synthesized from hypotaurine and elemental sulfur as reported [17,40]. Briefly, hypotaurine was dissolved in a mixture containing ethanol/NaOH and finely dispersed elemental sulfur. After 30 min boiling, the suspension was placed at 0 °C for 12 h. The obtained crystals were washed with ethanol and dissolved in water; thereafter, absolute ethanol was added. It was placed at 0 °C, and, after 12 h, the pure crystals were decanted.

### 4.2. Human Primary Cell Isolation and Culture

Human primary chondrocytes (HPCs) were obtained from OA patients undergoing joint replacement surgery treatment of the knee or hip, following previously established protocols [41]. Articular cartilage was aseptically harvested from femoral and tibial condyles as well as femoral heads. The cartilage tissue was finely minced and enzymatically digested using 1 mg/mL protease for 30 min, followed by treatment with 1 mg/mL type II collagenase at 37 °C for 4 h. The isolated HPCs were cultured at 37 °C in a humidified atmosphere containing 5% CO_2_ until they reached approximately 80% confluence. Cells were maintained in Dulbecco’s Modified Eagle Medium (DMEM) high-glucose (HyClone, Logan, UT, USA) supplemented with 1% L-glutamine, 1% penicillin/streptomycin (Sigma-Aldrich, Co. Saint Louis, MO, USA), 50 μg/mL gentamicin, and 10% fetal bovine serum (FBS). Informed consent was obtained from all donors, and the study received ethical approval from the Research Ethics Committee of Sapienza University of Rome (#290/07, 29 March 2007) and ASL Lazio 2 (#005605/2019, 3 March 2019).

### 4.3. Cell Treatment

Cells were left untreated (CTL) or stimulated with 10 ng/mL TNF-α or treated for the required time with different concentrations of Thiotaurine and with or without TNF-α stimulation. Experiments were independently repeated at least three times.

### 4.4. MTS Assay

To evaluate the cytotoxic effects of Thiotaurine on human primary chondrocytes (HPCs), a colorimetric assay based on MTS (3-(4,5-dimethylthiazol-2-yl)-5-(3-carboxymethoxyphenyl)-2-(4-sulfophenyl)-2H-tetrazolium) was conducted. In summary, HPCs were seeded at a density of 5 × 10^3^ cells per well in 96-well plates. The following day, cells were either left untreated (control, CTL) or exposed to various concentrations of Thiotaurine for 24, 48, and 72 h. At each time point, 100 µL of MTS reagent was added to each well. After a 3 h incubation period, absorbance was measured directly at 492 nm using a spectrophotometer.

### 4.5. RNA Extraction and Reverse Transcription

HPCs were either left untreated (CTL), stimulated with 10 ng/mL TNF-α for 30 min, or pre-treated for 1 h with 100 µM or 50 µM Thiotaurine before stimulation with 10 ng/mL TNF-α for 30 min. Total RNA was isolated from both treated and untreated cells using the Blood/Tissues Total RNA Extraction Kit (Fisher Molecular Biology, Trevose, PA, USA) and subsequently reverse-transcribed into cDNA using the Improm II reverse transcriptase (Promega Corporation, Madison, WI, USA), following the manufacturer’s protocol. The quality control of RNA was assessed using agarose gel and spectrophotometer measures, verifying the 260/280 ratio.

### 4.6. Quantitative Real-Time PCR

Quantitative real-time PCR (qRT-PCR) was performed using the ABI Prism 7300 system (Applied Biosystems, Thermo Fisher Scientific, Waltham, MA, USA). Amplification reactions were carried out with the SensimixPlus SYBR Master Mix. Primers (listed in Table 1) were synthesized by Bio-Fab Research and designed using Primer Express software version 1.4.0 (Applied Biosystems). Gene expression levels were calculated using the 2^−ΔΔCt^ method, with normalization to the 18S rRNA gene as an internal control [42].

### 4.7. Immunofluorescence Analysis

Immunofluorescence analysis was used to detect total p65 and phosphorylated p65 (p-p65) proteins. HPCs were seeded at a density of 5 × 10^3^ cells/cm^2^. The following day, cells were either left untreated (CTL), stimulated with 10 ng/mL TNF-α for 10 min, or pre-treated for 1 h with 100 µM or 50 µM Thiotaurine prior to stimulation with 10 ng/mL TNF-α for 10 min. Cells were then fixed with ethanol for 15 min at room temperature (RT) and permeabilized with 0.5% Triton X-100 in PBS for 10 min at RT. To block non-specific binding, cells were incubated with 3% bovine serum albumin (BSA) in PBS for 30 min. Subsequently, cells were incubated for 1 h at RT with primary antibodies against total p65 and phospho-p65 (Abcam, Cambridge, UK) (1:200 dilution). After washing with PBS, cells were incubated with Alexa Fluor 595-conjugated donkey anti-rabbit secondary antibody (Invitrogen, Thermo Fisher Scientific, Waltham, MA, USA) (1:400 dilution) for 1 h. Nuclei were counterstained with DAPI. All steps were carried out at room temperature. Images were acquired using a Leica DM IL LED microscope equipped with an AF6000 modular imaging system (Leica Microsystems, Milan, Italy).

### 4.8. ELISA

HPCs were seeded at a density of 1 × 10^5^ cells/cm^2^. The following day, cells were either left untreated (CTL), stimulated with 10 ng/mL TNF-α for 1 h, or pre-treated with 100 µM or 50 µM Thiotaurine for 1 h prior to stimulation with 10 ng/mL TNF-α for an additional hour. The concentrations of IL-6, IL-8, and IL-1β in the supernatants from both treated and untreated HPCs were quantified using ELISA kits (Fine Test ELISA, Fine Biotech Co., Ltd., Wuhan, China), following the manufacturer’s protocols. Absorbance was measured at 450 nm using a microplate reader (NeBiotech, Holden, MA, USA).

### 4.9. Persulphide Determination Assay

HPCs were plated at a density of 5 × 10^3^/cm^2^. The day after seeding, cells were left untreated (CTL) or treated for 24 h with 100 µM or 50 µM of Thiotaurine. Cells were washed in PBS, fixed in ethanol for 15 min at room temperature (RT), and permeabilized with 0.5% Triton-X 100 in PBS for 10 min at RT. Then, cellular proteins were blocked with 3% bovine serum albumin in PBS for 30 min. After blockage, cells were incubated with 1 mM EZ-Link™ Iodoacetyl-PEG2-Biotin (Thermo Fisher) for 2 h at 37 °C. Cells were washed with PBS and then incubated with Alexa Fluor 488-Streptavidin (Invitrogen, Thermo Fisher) (1:400) for 1 h at RT to visualize both sulfide and persulfide. Cells were washed and then stained with DAPI to visualize the nuclei.

To visualize sulfide only, cells were incubated with 30 mM DTT for 2 h at RT and then washed with PBS. The differences among the integrated fluorescence density values obtained in sulfide and persulfide wells and sulfide wells indicated the persulfide expression.

### 4.10. Densitometric Analysis

The free software ImageJ v1.54p (https://imagej.nih.gov/ij/, accessed on 1 July 2024) was used to perform densitometric analysis of protein expression. For each cell culture condition, the integrated fluorescence density values obtained in immunofluorescence experiments were considered.

### 4.11. Statistical Analysis

All data were collected from a minimum of three independent experiments, with each experiment conducted in duplicate or triplicate. Statistical analysis was performed using Prism 5.0 software (GraphPad Software, San Diego, CA, USA). Data distribution was evaluated for deviations from normality, and then two-way repeated measures ANOVA followed by Bonferroni post hoc tests were applied to evaluate differences between groups. A *p*-value of less than 0.05 was considered statistically significant.

## 5. Conclusions

As previously proposed, Thiotaurine represents a safe non-toxic storage form of H_2_S and a key intermediate in the biochemical routes of transport, storage, and release of sulfide. Given these properties, Thiotaurine could be considered as a H_2_S/sulfane sulfur donor with potential therapeutical applications. The health benefits of sulfur-based therapy, such as sulfur water springs, garlic, and cruciferous vegetables, have been recognized since ancient times. Notably, the common denominator in these remedies lies in their ability to supply H_2_S/sulfane sulfur, further supporting the therapeutic potential of sulfane sulfur compounds, such as Thiotaurine.

Future studies should aim to evaluate Thiotaurine’s effects in *in vivo* models of OA and explore its potential synergy with other redox-active compounds to fully understand its therapeutic potential. *In vivo* models would account for the complex interplay between multiple cell types, extracellular matrix components, and systemic factors influencing OA progression, which are not fully replicated *in vitro*. Moreover, proteomic approaches may help to identify specific protein targets of persulfidation in chondrocytes, deepening our understanding of the regulatory networks influenced by Thiotaurine. Thus, future investigations in animal models of OA and ultimately clinical studies will be critical to validate the protective and modulatory effects observed here, paving the way for potential clinical applications.

## Figures and Tables

**Table 1:** RT-PCR primer sequences.

Gene Accession Number	Primer Forward Primer Reverse
IL-6 NM_000600	5′-GATGGATGCTTCCAATCTG-3′ 5′-CTCTAGGTATACCTCAAACTCC-3′
IL-8 NM_000584	5′-GACATCAAAGAAGGACTTG-3′ 5′-GCCACAATTTCAGATCCTG-3′
IL-1β NM_000576	5′-ACAGAATCTCCGACCACCACTA-3′ 5′-TCCATGGCCACAACAACTGA-3′
18S NM_003286	5′-CGCCGCTAGAGGTGAAATTC-3′ 5′-CATTCTTGGCAAATGCTTTCG-3′

## Data Availability

Data is contained within the article and is available on request.
